# (*E*)-3-(4-Fluoro­phen­yl)-1-(2-nitro­phen­yl)prop-2-en-1-one

**DOI:** 10.1107/S160053681001809X

**Published:** 2010-05-22

**Authors:** Zhi-fang Pan

**Affiliations:** a Microscale Science Institute, Weifang University, Weifang 261061, People’s Republic of China

## Abstract

The title compound, C_15_H_10_F_N_O_3_, was prepared from 2-nitro­acetphenone and 4-fluoro­benzaldehyde by an Aldol condensation reaction. The dihedral angle formed by the two benzene rings is 67.37 (2)°. The crystal structure is stabilized by weak inter­molecular C—H⋯O and C—H⋯F hydrogen bonds.

## Related literature

For the biological activities of chalcones, see: Hsieh *et al.* (1998 ▶); Anto *et al.* (1994 ▶); De Vincenzo *et al.*(2000 ▶); Dimmock *et al.* (1998 ▶). For related structures, see: Fun *et al.* (2008 ▶); Guo *et al.* (2009 ▶).
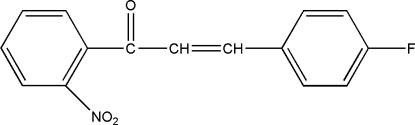


## Experimental

### 

#### Crystal data

C_15_H_10_FNO_3_
*M* *_r_* = 271.24
Monoclinic, 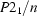

*a* = 7.7698 (16) Å
*b* = 17.072 (3) Å
*c* = 9.759 (2) Å
β = 96.72 (3)°
*V* = 1285.6 (5) Å^3^
*Z* = 4
Mo *K*α radiation
μ = 0.11 mm^−1^
*T* = 293 K
0.3 × 0.3 × 0.2 mm

#### Data collection

Bruker SMART CCD diffractometer
12293 measured reflections
2921 independent reflections
2218 reflections with *I* > 2σ(*I*)
*R*_int_ = 0.021

#### Refinement

*R*[*F* ^2^ > 2σ(*F* ^2^)] = 0.044
*wR*(*F* ^2^) = 0.141
*S* = 1.13
2921 reflections
181 parameters
H-atom parameters constrained
Δρ_max_ = 0.24 e Å^−3^
Δρ_min_ = −0.19 e Å^−3^

### 

Data collection: *SMART* (Bruker, 1997 ▶); cell refinement: *SAINT* (Bruker, 1997 ▶); data reduction: *SAINT*; program(s) used to solve structure: *SHELXS97* (Sheldrick, 2008 ▶); program(s) used to refine structure: *SHELXL97* (Sheldrick, 2008 ▶); molecular graphics: *SHELXTL* (Sheldrick, 2008 ▶); software used to prepare material for publication: *SHELXTL*.

## Supplementary Material

Crystal structure: contains datablocks global, I. DOI: 10.1107/S160053681001809X/lh5041sup1.cif

Structure factors: contains datablocks I. DOI: 10.1107/S160053681001809X/lh5041Isup2.hkl

Additional supplementary materials:  crystallographic information; 3D view; checkCIF report

## Figures and Tables

**Table 1:** Hydrogen-bond geometry (Å, °)

*D*—H⋯*A*	*D*—H	H⋯*A*	*D*⋯*A*	*D*—H⋯*A*
C4—H4*A*⋯O3^i^	0.93	2.57	3.500 (2)	177
C5—H5*A*⋯F1^ii^	0.93	2.54	3.396 (2)	153
C9—H9*A*⋯O3^iii^	0.93	2.57	3.411 (2)	150

Symmetry codes: (i) 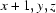; (ii) 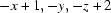; (iii) 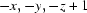.

**Fig. 1.**
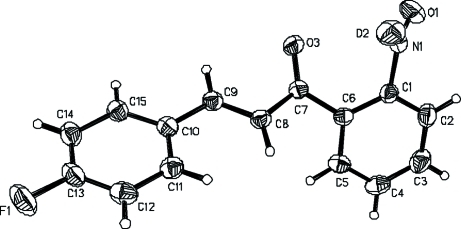
The molecular structure of the title compound with the atom-labeling scheme. Displacement ellipsoids are drawn at the 30% probability level.

## supplementary crystallographic information

### Comment

Among flavonoids, chalcones have been identified as interesting compounds having multiple biological activities which include antiinflammatory (Hsieh *et al.*,1998) and antioxidant (Anto *et al.*,1994). Of particular interest, the effectiveness of chalcones against cancer has been investigated (De Vincenzo *et al.*,2000; Dimmock *et al.*,1998). As part of our search for new biologically active compounds we synthesized the title compound (I) and report its crystal structure herein.

In the title molecule (Fig. 1) the dihedral angle formed by the two benzene rings is 67.44 (3)°. The nitro group is twisted from the attached benzene ring forming a dihedral angle of 53.73 (5)°. All of the bond lengths and bond angles are in normal ranges and comparable to those in related structures (Fun *et al.*,2008; Guo *et al.*,2009). The crystal structure is stabilized by weak intermolecular C—H···O and C—H···F hydrogen bonds.

### Experimental

A mixture of 2-nitroacetphenone (0.02 mol), 4-fluorobenzaldehyde (0.02 mol) and 8% NaOH(5 ml) was stirred in ethanol(30 ml) for 4 h to afford the title compound (yield 65%). Single crystals suitable for X-ray measurements were obtained by recrystallization from ethyl acetate at room temperature.

### Refinement

H atoms were positioned geometrically and allowed to ride on their parent atoms, with C—H distances of 0.93 Å, and with *U*_iso_(H) = 1.2*U*_eq_ of the parent atoms.

### Crystal data

**Table:**

C_15_H_10_FNO_3_	*F*(000) = 560
*M**_r_* = 271.24	*D*_x_ = 1.401 Mg m^−^^3^
Monoclinic, *P*2_1_/*n*	Mo *K*α radiation, λ = 0.71073 Å
Hall symbol: -P 2yn	Cell parameters from 2218 reflections
*a* = 7.7698 (16) Å	θ = 3.2–27.5°
*b* = 17.072 (3) Å	µ = 0.11 mm^−^^1^
*c* = 9.759 (2) Å	*T* = 293 K
β = 96.72 (3)°	Bar, colourless
*V* = 1285.6 (5) Å^3^	0.3 × 0.3 × 0.2 mm
*Z* = 4	

### Data collection

**Table:**

Bruker SMART CCD diffractometer	2218 reflections with *I* > 2σ(*I*)
Radiation source: fine-focus sealed tube	*R*_int_ = 0.021
graphite	θ_max_ = 27.5°, θ_min_ = 3.2°
φ and ω scans	*h* = −9→10
12293 measured reflections	*k* = −22→22
2921 independent reflections	*l* = −12→12

### Refinement

**Table:**

Refinement on *F*^2^	Primary atom site location: structure-invariant direct methods
Least-squares matrix: full	Secondary atom site location: difference Fourier map
*R*[*F*^2^ > 2σ(*F*^2^)] = 0.044	Hydrogen site location: inferred from neighbouring sites
*wR*(*F*^2^) = 0.141	H-atom parameters constrained
*S* = 1.13	*w* = 1/[σ^2^(*F*_o_^2^) + (0.0695*P*)^2^ + 0.2079*P*] where *P* = (*F*_o_^2^ + 2*F*_c_^2^)/3
2921 reflections	(Δ/σ)_max_ < 0.001
181 parameters	Δρ_max_ = 0.24 e Å^−^^3^
0 restraints	Δρ_min_ = −0.19 e Å^−^^3^

### Special details

**Table:**

Geometry. All esds (except the esd in the dihedral angle between two l.s. planes) are estimated using the full covariance matrix. The cell esds are taken into account individually in the estimation of esds in distances, angles and torsion angles; correlations between esds in cell parameters are only used when they are defined by crystal symmetry. An approximate (isotropic) treatment of cell esds is used for estimating esds involving l.s. planes.
Refinement. Refinement of *F*^2^ against ALL reflections. The weighted *R*-factor wR and goodness of fit *S* are based on *F*^2^, conventional *R*-factors *R* are based on *F*, with *F* set to zero for negative *F*^2^. The threshold expression of *F*^2^ > σ(*F*^2^) is used only for calculating *R*-factors(gt) etc. and is not relevant to the choice of reflections for refinement. *R*-factors based on *F*^2^ are statistically about twice as large as those based on *F*, and *R*- factors based on ALL data will be even larger.

### Fractional atomic coordinates and isotropic or equivalent isotropic displacement parameters (Å^2^)

**Table:**

	*x*	*y*	*z*	*U*_iso_*/*U*_eq_	
O3	0.08601 (15)	0.10025 (8)	0.43283 (13)	0.0659 (4)	
C7	0.22626 (19)	0.11601 (9)	0.49650 (15)	0.0449 (3)	
C9	0.18075 (19)	0.04525 (9)	0.70546 (15)	0.0469 (3)	
H9A	0.0871	0.0226	0.6517	0.056*	
C6	0.36095 (18)	0.15551 (8)	0.42269 (14)	0.0418 (3)	
C8	0.27359 (19)	0.09685 (9)	0.64309 (15)	0.0472 (4)	
H8A	0.3689	0.1209	0.6924	0.057*	
C15	0.1386 (2)	−0.05036 (9)	0.88492 (16)	0.0495 (4)	
H15A	0.0725	−0.0794	0.8173	0.059*	
F1	0.27941 (18)	−0.06060 (9)	1.24775 (11)	0.0933 (4)	
N1	0.13799 (19)	0.23833 (8)	0.29207 (17)	0.0589 (4)	
C10	0.21056 (19)	0.02052 (9)	0.84967 (15)	0.0449 (3)	
C11	0.3057 (2)	0.06445 (10)	0.95326 (16)	0.0522 (4)	
H11A	0.3536	0.1122	0.9317	0.063*	
C1	0.31757 (19)	0.20928 (9)	0.31805 (16)	0.0471 (4)	
C5	0.5339 (2)	0.13342 (10)	0.44662 (16)	0.0521 (4)	
H5A	0.5685	0.0977	0.5165	0.063*	
C14	0.1637 (2)	−0.07817 (11)	1.01808 (18)	0.0569 (4)	
H14A	0.1179	−0.1261	1.0408	0.068*	
C12	0.3287 (2)	0.03713 (12)	1.08766 (17)	0.0595 (5)	
H12A	0.3911	0.0661	1.1574	0.071*	
C13	0.2572 (2)	−0.03371 (12)	1.11546 (16)	0.0593 (5)	
C4	0.6559 (2)	0.16380 (12)	0.36778 (19)	0.0634 (5)	
H4A	0.7714	0.1487	0.3857	0.076*	
O2	0.07277 (18)	0.26603 (9)	0.38907 (18)	0.0830 (5)	
C2	0.4367 (2)	0.23925 (11)	0.2374 (2)	0.0643 (5)	
H2A	0.4026	0.2745	0.1667	0.077*	
C3	0.6067 (3)	0.21593 (13)	0.2637 (2)	0.0702 (5)	
H3A	0.6888	0.2356	0.2106	0.084*	
O1	0.0655 (2)	0.23414 (11)	0.17437 (17)	0.0930 (5)	

### Atomic displacement parameters (Å^2^)

**Table:**

	*U*^11^	*U*^22^	*U*^33^	*U*^12^	*U*^13^	*U*^23^
O3	0.0545 (7)	0.0779 (9)	0.0612 (7)	−0.0237 (6)	−0.0098 (5)	0.0163 (6)
C7	0.0435 (8)	0.0443 (8)	0.0459 (8)	−0.0029 (6)	0.0003 (6)	0.0010 (6)
C9	0.0446 (8)	0.0472 (8)	0.0477 (8)	−0.0016 (6)	0.0013 (6)	−0.0025 (6)
C6	0.0411 (7)	0.0437 (7)	0.0396 (7)	−0.0021 (6)	0.0009 (5)	−0.0065 (6)
C8	0.0424 (7)	0.0526 (9)	0.0455 (8)	−0.0057 (6)	0.0006 (6)	−0.0017 (6)
C15	0.0508 (8)	0.0491 (8)	0.0488 (8)	0.0039 (6)	0.0063 (7)	0.0005 (6)
F1	0.1009 (9)	0.1298 (11)	0.0478 (6)	0.0207 (8)	0.0028 (6)	0.0285 (6)
N1	0.0533 (8)	0.0473 (8)	0.0755 (10)	0.0011 (6)	0.0049 (7)	0.0163 (7)
C10	0.0432 (7)	0.0503 (8)	0.0417 (7)	0.0059 (6)	0.0076 (6)	−0.0004 (6)
C11	0.0486 (8)	0.0571 (9)	0.0515 (8)	−0.0001 (7)	0.0084 (7)	−0.0037 (7)
C1	0.0458 (8)	0.0396 (7)	0.0560 (9)	−0.0011 (6)	0.0070 (6)	0.0006 (6)
C5	0.0474 (8)	0.0621 (10)	0.0456 (8)	0.0040 (7)	0.0002 (6)	−0.0042 (7)
C14	0.0590 (9)	0.0554 (9)	0.0577 (9)	0.0109 (7)	0.0126 (8)	0.0124 (7)
C12	0.0538 (9)	0.0791 (12)	0.0448 (8)	0.0068 (8)	0.0020 (7)	−0.0115 (8)
C13	0.0555 (9)	0.0819 (12)	0.0411 (8)	0.0204 (9)	0.0075 (7)	0.0121 (8)
C4	0.0413 (8)	0.0821 (13)	0.0678 (11)	0.0007 (8)	0.0105 (8)	−0.0142 (9)
O2	0.0623 (8)	0.0779 (10)	0.1116 (12)	0.0149 (7)	0.0225 (8)	−0.0109 (8)
C2	0.0669 (11)	0.0554 (10)	0.0734 (12)	−0.0025 (8)	0.0199 (9)	0.0147 (8)
C3	0.0601 (11)	0.0744 (12)	0.0809 (13)	−0.0075 (9)	0.0287 (10)	0.0056 (10)
O1	0.0797 (10)	0.1129 (13)	0.0809 (10)	0.0104 (9)	−0.0132 (8)	0.0361 (9)

### Geometric parameters (Å, °)

**Table:**

O3—C7	1.2195 (18)	C10—C11	1.399 (2)
C7—C8	1.472 (2)	C11—C12	1.384 (2)
C7—C6	1.498 (2)	C11—H11A	0.9300
C9—C8	1.330 (2)	C1—C2	1.382 (2)
C9—C10	1.462 (2)	C5—C4	1.390 (2)
C9—H9A	0.9300	C5—H5A	0.9300
C6—C1	1.385 (2)	C14—C13	1.359 (3)
C6—C5	1.389 (2)	C14—H14A	0.9300
C8—H8A	0.9300	C12—C13	1.371 (3)
C15—C14	1.376 (2)	C12—H12A	0.9300
C15—C10	1.393 (2)	C4—C3	1.371 (3)
C15—H15A	0.9300	C4—H4A	0.9300
F1—C13	1.3619 (18)	C2—C3	1.375 (3)
N1—O2	1.220 (2)	C2—H2A	0.9300
N1—O1	1.221 (2)	C3—H3A	0.9300
N1—C1	1.475 (2)		
			
O3—C7—C8	123.58 (14)	C2—C1—C6	122.96 (15)
O3—C7—C6	119.14 (13)	C2—C1—N1	117.45 (15)
C8—C7—C6	117.27 (12)	C6—C1—N1	119.56 (13)
C8—C9—C10	126.77 (14)	C6—C5—C4	121.00 (16)
C8—C9—H9A	116.6	C6—C5—H5A	119.5
C10—C9—H9A	116.6	C4—C5—H5A	119.5
C1—C6—C5	116.81 (14)	C13—C14—C15	118.23 (17)
C1—C6—C7	121.96 (13)	C13—C14—H14A	120.9
C5—C6—C7	120.89 (14)	C15—C14—H14A	120.9
C9—C8—C7	120.32 (14)	C13—C12—C11	118.32 (16)
C9—C8—H8A	119.8	C13—C12—H12A	120.8
C7—C8—H8A	119.8	C11—C12—H12A	120.8
C14—C15—C10	121.09 (16)	C14—C13—F1	118.22 (18)
C14—C15—H15A	119.5	C14—C13—C12	123.39 (15)
C10—C15—H15A	119.5	F1—C13—C12	118.40 (17)
O2—N1—O1	124.40 (16)	C3—C4—C5	120.24 (16)
O2—N1—C1	117.87 (15)	C3—C4—H4A	119.9
O1—N1—C1	117.71 (16)	C5—C4—H4A	119.9
C15—C10—C11	118.74 (14)	C3—C2—C1	118.66 (17)
C15—C10—C9	117.76 (14)	C3—C2—H2A	120.7
C11—C10—C9	123.49 (15)	C1—C2—H2A	120.7
C12—C11—C10	120.21 (16)	C4—C3—C2	120.31 (16)
C12—C11—H11A	119.9	C4—C3—H3A	119.8
C10—C11—H11A	119.9	C2—C3—H3A	119.8

### Hydrogen-bond geometry (Å, °)

**Table:**

*D*—H···*A*	*D*—H	H···*A*	*D*···*A*	*D*—H···*A*
C4—H4A···O3^i^	0.93	2.57	3.500 (2)	177
C5—H5A···F1^ii^	0.93	2.54	3.396 (2)	153
C9—H9A···O3	0.93	2.51	2.836 (2)	101
C9—H9A···O3^iii^	0.93	2.57	3.411 (2)	150

Symmetry codes: (i) *x*+1, *y*, *z*; (ii) −*x*+1, −*y*, −*z*+2; (iii) −*x*, −*y*, −*z*+1.
